# Myocarditis and pericarditis in individuals exposed to the Ad26.COV2.S, BNT162b2 mRNA, or mRNA-1273 SARS-CoV-2 vaccines

**DOI:** 10.3389/fcvm.2023.1210007

**Published:** 2023-11-24

**Authors:** Manan Pareek, Pasquale Sessa, Paolo Polverino, Francesco Sessa, Kristian Hay Kragholm, Maurizio Sessa

**Affiliations:** ^1^ Center for Translational Cardiology and Pragmatic Randomized Trials, Copenhagen University Hospital—Herlev and Gentofte, Hellerup, Denmark; ^2^ Department of Cardiology, Copenhagen University Hospital—Rigshospitalet, Copenhagen, Denmark; ^3^ Emergency Department, San Camillo—Forlanini Hospital Rome, Rome, Italy; ^4^ Department of Experimental and Clinical Medicine, University of Florence, Florence, Italy; ^5^ Department of Cardiology, Aalborg University Hospital, Aalborg, Denmark; ^6^ Department of Drug Design and Pharmacology, University of Copenhagen, Copenhagen, Denmark

**Keywords:** myocarditis, pericarditis, coronavirus, vaccination, VAERS, Vaccine Adverse Event Reporting System, mRNA vaccine against SARS-CoV-2

## Abstract

**Importance:**

There is a high level of public and professional interest related to potential safety issues of the COVID-19 vaccines; however, no serious adverse cardiovascular events were reported in phase 3 randomized controlled trials of their safety and efficacy. Moreover, none of the case series from the United States (US) of these potential complications have been population-based.

**Objectives:**

To estimate the reporting rates of myocarditis and pericarditis in the US using the Vaccine Adverse Event Reporting System (VAERS), and to assess if these adverse events were disproportionally reported among the different COVID-19 vaccines.

**Design, setting, and participants:**

All cases of myocarditis and pericarditis from VAERS reported up to July 28, 2021.

**Exposure:**

Single-dose Ad26.COV2.S, BNT162b2 mRNA, or mRNA-1273 SARS-CoV-2 vaccinations.

**Main outcomes and measures:**

Reporting rates were computed by dividing the total number of cases of myocarditis and pericarditis (combined) by the total number of vaccine doses administered. Disproportionality analyses were performed to evaluate disproportional reporting of myocarditis and pericarditis for the Ad26.COV2.S and mRNA-1273 vaccines vs. the BNT162b2 mRNA vaccine.

**Results:**

By July 28, 2021, 1392, 699, and 68 cases of myocarditis or pericarditis had been reported out of 1.91, 1.38, and 1.33 million administered doses of the BNT162b2 mRNA, mRNA-1273, and Ad26.COV2.S COVID-19 vaccines, respectively. Median times to event were 3 days, 3 days, and 9 days for the BNT162b2 mRNA, mRNA-1273, and Ad26.COV2.S COVID-19 vaccines. The reporting rates for myocarditis or pericarditis were 0.00073 (95% confidence interval, 95% CI 0.00069–0.00077), 0.00051 (95% CI 0.00047–0.00055), and 0.00005 events per dose (95% CI 0.00004–0.00006) for the BNT162b2 mRNA, mRNA-1273, and Ad26.COV2.S COVID-19 vaccines, respectively. Myocarditis and pericarditis were disproportionally reported following the BNT162b2 mRNA vaccine when compared with the other vaccines, using both disproportionality measures.

**Conclusions and relevance:**

We found reporting rates of myocarditis and pericarditis to be less than 0.1% after COVID-19 vaccination. Rates were highest for the BNT162b2 mRNA vaccine, followed by the mRNA-1273 and Ad26.COV2.S, respectively. However, the reporting rates of myocarditis and pericarditis secondary to vaccination remains less common than those seen for SARS-CoV-2 infection.

## 1. Introduction

Myocarditis is inflammation of the cardiac muscle, while pericarditis is a pericardial inflammatory syndrome (1–3). Myocarditis and pericarditis share common etiologies, and overlapping forms are often encountered (4). While both conditions are most frequently triggered by viral infection, now including severe acute respiratory syndrome coronavirus 2 (SARS-CoV-2) (5), cases have been reported following the BNT162b2-mRNA (Pfizer-BioNTech) and mRNA-1273 (Moderna) coronavirus disease 2019 (COVID-19) vaccines (6). There is a high level of public and professional interest related to potential safety issues of the COVID-19 vaccines, but no serious adverse cardiovascular events were reported in phase 3 randomized controlled trials of their safety and efficacy (7–9). Although rates of myocarditis or pericarditis following COVID-19 vaccines appear to be higher than expected when compared with the background population, early case series from the United States (US) of these potential complications were not population-based (10), and reported studies using data from the Vaccine Adverse Event Reporting System (VAERS) have not directly compared the three most commonly administered vaccines (11–16). Therefore, we aimed to describe the reporting rates of myocarditis and pericarditis in the US, assess disproportionality in reporting myocarditis or pericarditis among the different vaccines, and describe the characteristics of individuals who developed these conditions.

## 2. Methods

### 2.1. Data sources

Using VAERS, we retrieved all cases of myocarditis and pericarditis following single-dose Ad26.COV2.S, BNT162b2 mRNA, or mRNA-1273 SARS-CoV-2 vaccinations reported up to July 28, 2021. Medical Dictionary for Regulatory Activities (MedDRA) preferred terms used to retrieve the cases from VAERS were myocarditis, pericarditis, viral myocarditis, and viral pericarditis.

### 2.2. Case-by-case analysis

A descriptive analysis of the demographic and clinical characteristics of the cases was performed. Age and sex stratified by vaccine type were presented in a density plot and a bar chart, respectively. A case-by-case analysis was performed by two researchers (PP and PS) which included an evaluation of risk factors for myocarditis and pericarditis (17, 18), the medical confirmation of the diagnosis by laboratory analyses and/or imaging, and an analysis of the narrative description of the case provided by the healthcare providers. Based on the results of the case-by-case analysis, we classified the cases as having/not having a validated diagnosis and has having/not having concurrent risk factors for myocarditis and pericarditis. During the case-by-case analysis, we screened for duplicates and cases that required obvious exclusion. This approach has been extensively used in VAERS and other spontaneous reporting databases (19–24).

### 2.3. Disproportionality analyses

Reporting rates were computed by dividing the total number of cases of myocarditis and pericarditis (combined) by the total number of vaccine doses administered. Reporting rate ratios were computed by dividing the reporting rates for myocarditis and pericarditis (combined) of the Ad26.COV2.S and mRNA-1273 vaccines by the reporting rate of the BNT162b2 mRNA vaccine. The 95% confidence intervals (95% CI) of the reporting rates and reporting rate ratios were also computed.

Disproportionality analyses were performed to evaluate disproportional reporting of myocarditis and pericarditis for the Ad26.COV2.S and mRNA-1273 vaccines vs. the BNT162b2 mRNA vaccine. The reporting odds ratio (ROR) and Empirical Bayes Geometric Mean (EBGM) were used as disproportionality measures, the formulas of which are presented in Table 1. An additional disproportionality analysis was performed only using cases that through case-by-case assessment were found to have a validated diagnosis and for which no risk factors were reported.

**Table 1:** Formulas for computing the reporting odds ratio (ROR), the standard error for the ROR, the empirical Bayes geometric mean (EBGM), and the 95% confidence interval for the EBGM.

Groups	Cases of interest (e.g., myocarditis or pericarditis)	Other cases
Moderna or Janssen Covid-19 vaccines	A	C
Pfizer-BioNTech Covid-19 vaccines	B	D
* *	Table legend	* *
Disproportionality measures	$\begin{array}{c} \mathrm{ROR}\;=\;(a/c)\;/\;(b/d)\\ \mathrm{Standard}\;\mathrm{error}\;(\mathrm{Ln}\;\mathrm{ROR})=\\ \sqrt{\left(\frac{1}{a}+\frac{1}{b}+\frac{1}{c}+\frac{1}{d}\right)} \end{array}$	$\begin{array}{c} \mathrm{EGBM}=\frac{a(a+b+c+d)}{\left(a+c)(a+b\right)}\\ 95\text{\%}\;\mathrm{Confidence}\;\mathrm{Interval}\;(\mathrm{EGBM})\\ =\mathrm{EGBM}*e\frac{\pm 1.645}{\sqrt{c_{\mathrm{ij}}+1}}\\ \mathrm{Where}\;C_{\mathrm{ij}}=\left(a+b)(a+c\right)/\left(a+b+c+d\right) \end{array}$

We considered a diagnosis corroborated by laboratory analysis and/or imaging if at least one of the following had been described:

1) Troponin concentration above the 99th percentile upper reference limit;
2) Electrocardiogram with typical alterations observed in cases of myocarditis or pericarditis, e.g., diffuse ST-segment elevations and PR-segment depressions;
3) Findings on magnetic resonance imagining or echocardiogram supporting the diagnosis of myocarditis or pericarditis;
4) Endomyocardial biopsy results supporting the diagnosis of myocarditis;

Additionally, we ensured that coronary angiography or CT-angiography did not support a diagnosis of obstructive coronary artery disease or pulmonary embolism.

## 3. Results

### 3.1. Descriptive analysis

By July 28, 2021, 1392, 699, and 68 cases of myocarditis or pericarditis had been reported out of 1.91, 1.38, and 1.33 million administered doses of the BNT162b2 mRNA, mRNA-1273, and Ad26.COV2.S COVID-19 vaccines. In all, 815 of 1392 (58%) and 369 of 699 (53%) cases of myocarditis or pericarditis followed the second dose of the BNT162b2 mRNA and mRNA-1273 COVID-19 vaccines, respectively.

The median times to event were 3 days (interquartile range, IQR 1–10 days), 3 days (IQR 2–13), and 9 days (IQR 2–25) for the BNT162b2 mRNA, mRNA-1273, and Ad26.COV2.S COVID-19 vaccines. Nine-hundred and seventy-five, 500, and 40 individuals required hospitalization for myocarditis or pericarditis. There were 10, 9, and 2 fatal cases, respectively.

### 3.2. Case-by-case analysis

In the case-by-case analysis, we identified 307 duplicates and 1 case for which it was claimed that no COVID-19 vaccine had been received. Therefore, these cases were excluded from further analyses.

**Figure 1:**
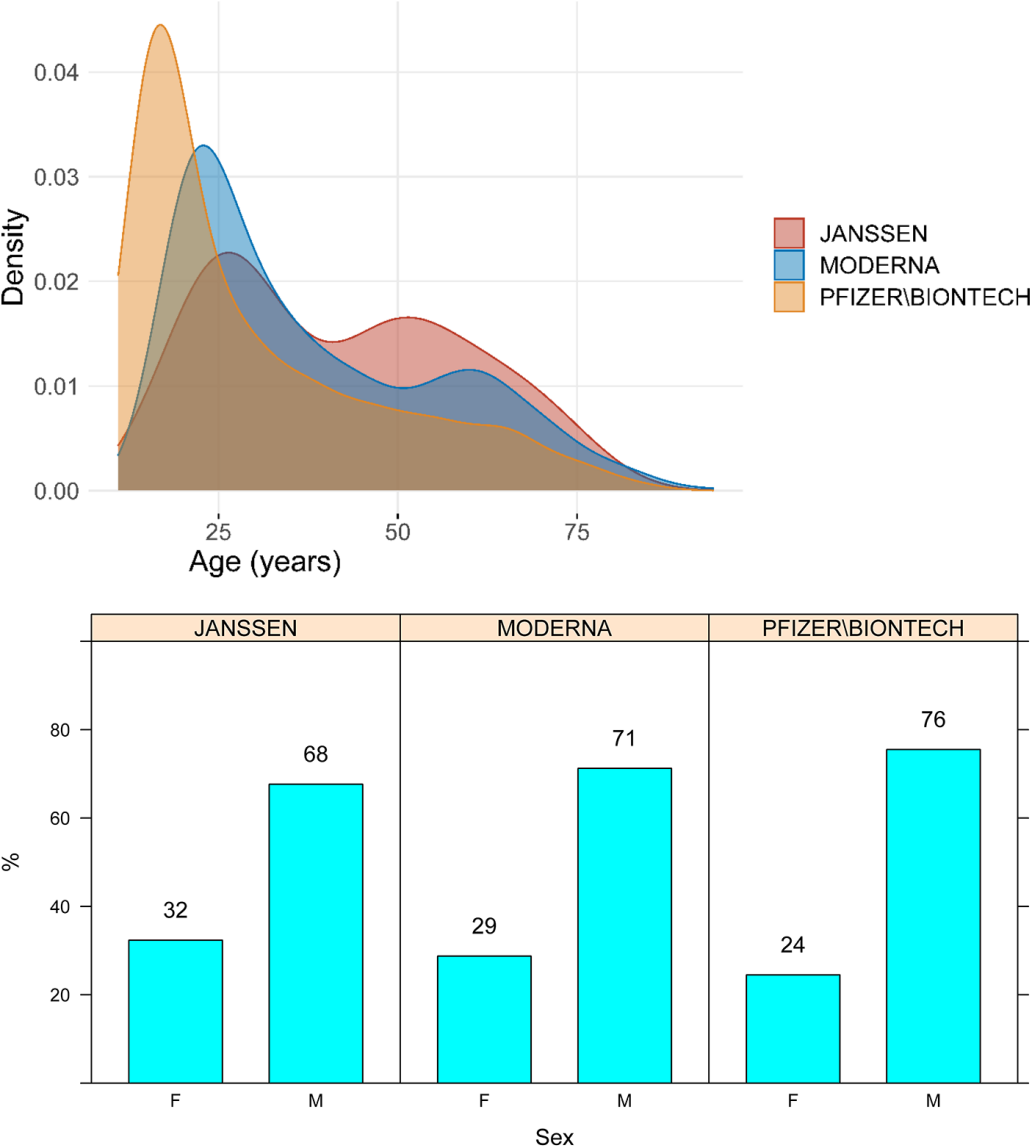
Age and sex distribution of cases of myocarditis and pericarditis by vaccine manufacturer. M, male; F, Female.

Median age of the cases was 27 years (IQR, 18–45), and 74% were male. The age and sex distributions of cases stratified by vaccine manufacturer are presented in Figure 1.

A total of 3,662 examinations were performed by healthcare providers to confirm the diagnosis of myocarditis or pericarditis, and to exclude the concurrent presence of clinical conditions that may have overlapping signs and symptoms. None of the cases reported a medical history of birth defects.

Forty-eight percent of the diagnoses were confirmed by laboratory analyses or imaging, without the presence of risk factors. In 7%, the diagnosis was supported by these ancillary tests, but risk factors had been reported. In 40% of the cases, the diagnosis was not confirmed by laboratory analyses or imaging, and risk factors were not reported. Finally, in 5%, the diagnosis was not confirmed by ancillary tests, but risk factors were reported.

Representative examples of clusters of cases with and without a diagnosis validated by laboratory analyses or medical imaging, and with or without concurrent risk factors are provided in Table 2. All cases evaluated are provided in Supplementary Table S1.

**Table 2:** Representative examples for clusters of similar cases of myocarditis and pericarditis following pfizer-bioNTech, moderna, and janssen COVID-19 vaccines exposure.

Cluster	Case reports
The diagnosis was medically confirmed, laboratory analysis supported the diagnosis of myocarditis/pericarditis, and risk factors were not reported	Age: 31 Sex: Male State: Minnesota Narrative description of the case: The patient reports a severe dull ache pain radiating in the left arm that’s been intermittent over the past week and associated tightness in neck/chest & diaphoresis. The patient received the 2nd Moderna shot the week before the occurrence of the first signs and symptoms and described feeling extensive discomfort 24 h following the vaccination and, then started feeling the abovementioned symptoms. The electrocardiogram showed sinus rhythm and ST elevations in V3–V6 and in the inferior leads (II, III, and aVF). Troponins were 8.32 at the admission and peaked to 13.58 after 3 h. At the admission, C-reactive protein was 6.1 and erythrocyte Sedimentation Rate 17. Chest x-ray was clear. Cardiac catheterization was performed to rule out obstructive coronary artery disease. Following this exam, Magnetic Resonance Imaging was performed and it confirmed myocarditis. The patient improved and was ultimately discharged in a week with a clinical follow-up in 1 month. Exams: Magnetic Resonance Imaging of the heart: findings concerning acute myocarditis edema & sub-epicardial delayed enhancement of left ventricle inferior & lateral walls at the base (ejection fraction 54%). Risk factors: none.
Laboratory analyses supported the diagnosis of myocarditis/pericarditis however, risk factors for myocarditis/pericarditis were reported.	Age: 76 Sex: Female State: Oregon Narrative description of the case: Acute myocarditis. Exams: Patient initially presenting with recurrent atypical chest pain, now third episode in the past week with similar presenting symptoms. Recently hospitalized for suspected non-ST elevation myocardial infarction, with coronary angiogram at that time demonstrating non-obstructive coronary artery disease (1/4/2021). Readmitted 27/4/2021 with recurrent chest pain and started on antianginal therapy with sublingual nitroglycerin and isosorbide mononitrate which were ineffective, subsequently resulting in current readmission (29/4/2021). Given mild persistent troponin elevation (200), cardiology consultation was obtained. Repeat transthoracic echocardiogram (30/4/2021) demonstrating interval improvement in ejection fraction with resolution of previously demonstrated wall motion abnormalities, ejection fraction is now 65%. Cardiac magnetic resonance was obtained which demonstrated abnormal T1 and T2 signal changes in the left ventricle consistent with myocarditis, notably no washing abnormalities were present. Per cardiology, likely resolving viral myocarditis. No further cardiac risk ratification indicated at this time. Cardiology advised symptomatic management of intermittent chest pain with hydrocodone/acetaminophen and avoidance of nonsteroidal medications. Patient was extensively counseled on the etiology of her chest pain, with emphasis on reassuring findings from recent coronary angiogram and cardiac MRI that chest pain does not appear to be due to acute coronary syndrome/ischemic heart disease. Sublingual nitroglycerin and isosorbide mononitrate were discontinued, and she was restarted on prior dose losartan for initial medical therapy with angiotensin-converting enzyme inhibitors given non-ischemic cardiomyopathy. *Cardiovascular magnetic resonance 30/4/2021:* 1. Abnormal T1 and T2 signal changes in the left ventricle with at least one focus of subepicardial enhancement involving the mid inferolateral segment, compatible with myocarditis. There are no wall motion abnormalities on the current exam to suggest stress cardiomyopathy. 2. Normal left-ventricular systolic function, ejection fraction 67%. **Risk factors:** concurrent cardiovascular disorders, hypothyroidism, and metastatic breast, and lung cancer.
The diagnosis was not medically confirmed by laboratory analyses but no risk factors for myocarditis/pericarditis were concurrently reported.	Age: 14 Sex: Male State: Maryland Narrative description of the case: The patient presented to the emergency room with a severe unrelenting chest pain beginning abruptly 4 days after receiving first dose of Pfizer COVID19 vaccine. He was diagnosed at the emergency room with pericarditis and discharged with ibuprofen. Chest pain has gradually improved over past 6 days though is still intermittently present. Exams: Electrocardiogram and chest x-ray were done at emergency room were reported to the parent as normal. Laboratory tests: Complete Blood Count: normal, C-reactive protein: normal, troponins: normal. Risk factors: none.
	Age: 17 Sex: Male State: California Narrative description of the case: A **co**uple days after my son (17 years old) got the 2nd shot he was having a pressure in his chest and left arm so we rushed him to the hospital. When we got to the hospital blood test show also liver inflammation they hospitalized him right away. He was there 3 days and just got released. Now he need to be under care with medication and visit to a heart cardiology doctor every few days for tests. He cannot do any activity (per to the doctor including computer games that can raise his heart rate). Exams: Inflammation of the heart, many test was takes. Risk factors: none.
The diagnosis was not medically confirmed by laboratory analyses and risk factors for myocarditis/pericarditis were concurrently reported.	Age: 39 Sex: Male State: South Carolina Narrative description of the case: 1/5/21 started with fever, severe body aches, shaking from being cold even bundled with electric blanket, nausea, and vomiting. That lasted through 1/8/2021. On 1/7 I started having trouble with taking a deep breath. Chest would get very tight and hurt when I would take a big breath, bend forward, or lay back. I went to express care and they could not rule out pericarditis. Told me to go to the emergency room for further work up to rule out spontaneous pulmonary embolism or pericarditis. Exams: 1/9 electrocardiogram and chest x-ray, both looked good. They did not have an echo or a computed tomography scanner. I have been taking 600 mg ibuprofen every 6 h (around the clock) to clear the inflammation and as of this morning 1/10 I am feeling 95% better! I will call my rheumatologist tomorrow when they open to see if he recommends echocardiogram/computed tomography to check my heart. Risk factors: Systemic lupus erythematosus.

### 3.3. Reporting rates, reporting rate ratios, and disproportionality analyses

The reporting rates for myocarditis/pericarditis were 0.00073 (95% confidence interval, 95% CI 0.00069–0.00077), 0.00051 (95% CI 0.00047–0.00055), and 0.00005 events per dose (95% CI 0.00004–0.00006) for the BNT162b2 mRNA, mRNA-1273, and Ad26.COV2.S COVID-19 vaccines, respectively. The reporting rate ratios for myocarditis or pericarditis were 1.44 (95% CI 1.31–1.58; BNT162b2 mRNA vs. mRNA-1273), 18.87 (95% CI 10.57–33.72; mRNA-1273 vs. Ad26.COV2.S), and 14.26 (95% CI 11.18–18.20; BNT162b2 mRNA vs. Ad26.COV2.S).

**Figure 2:**
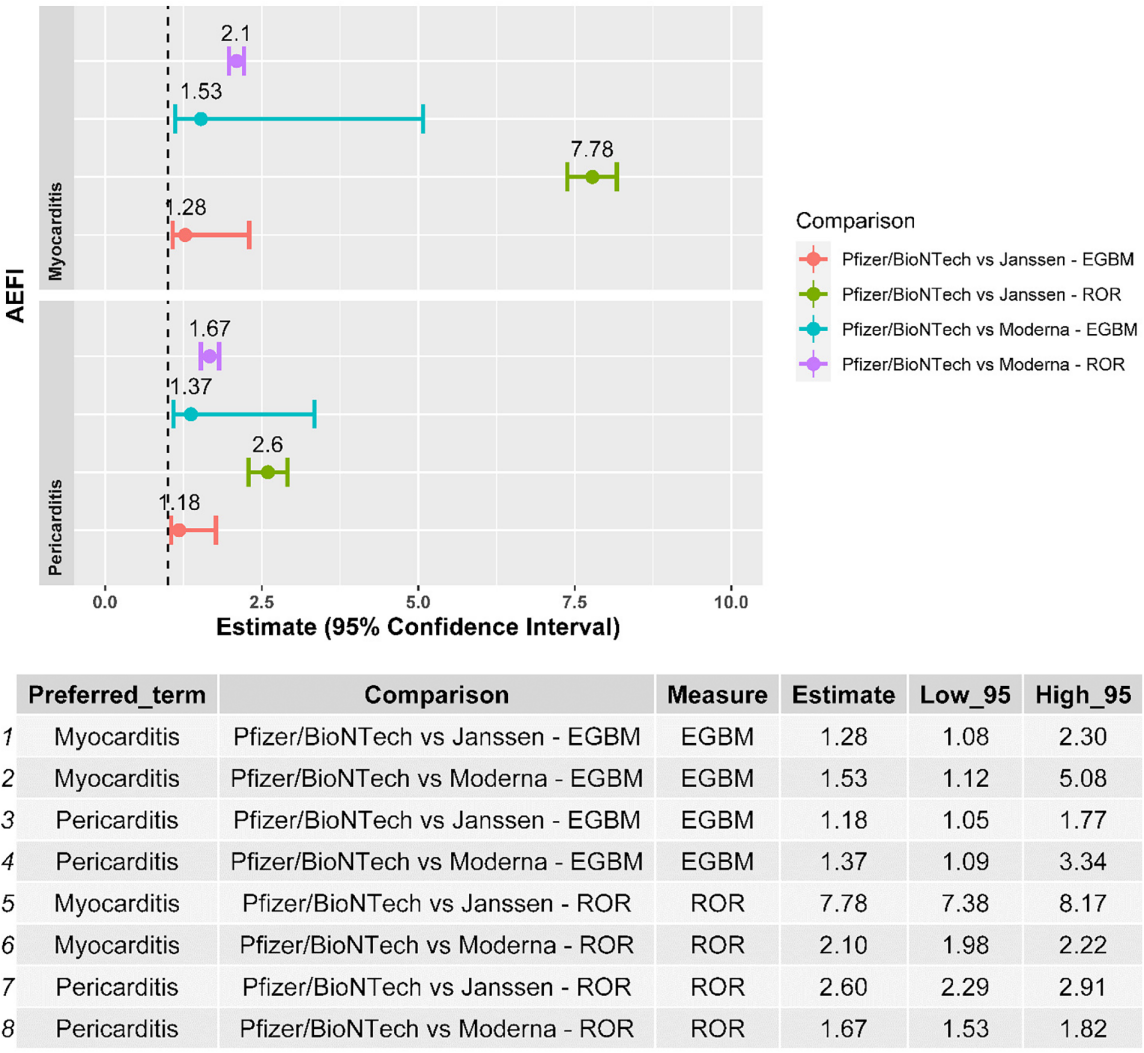
Forest plot of the results of the disproportionality analysis*.* ROR, reporting odds ratio; EBGM, empirical Bayes geometric mean; AEFI, adverse event following immunization.

**Figure 3:**
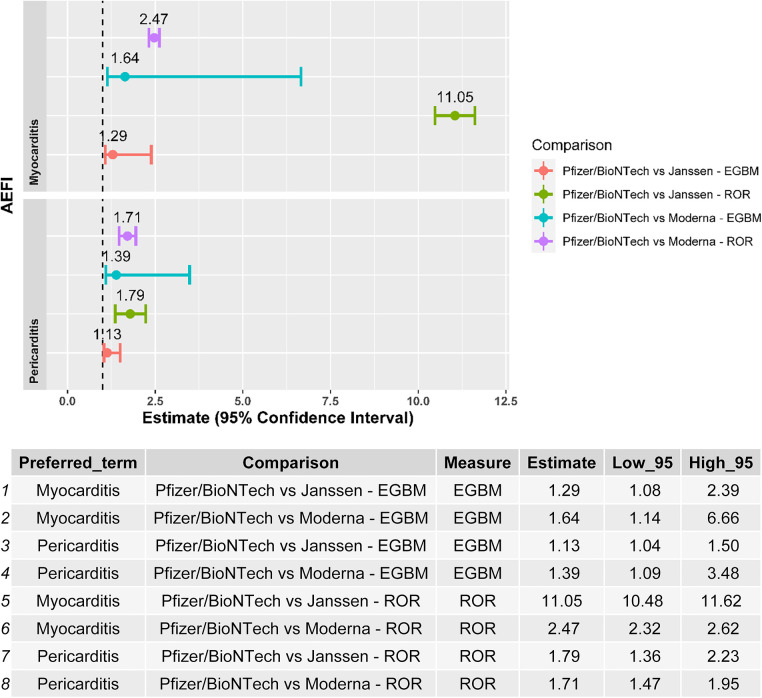
Forest plot of the results of the disproportionality analysis performed with validated cases by laboratory analysis/medical imaging of myocarditis and pericarditis*.* ROR, reporting odds ratio; EBGM, empirical Bayes geometric mean; AEFI, adverse event following immunization.

Myocarditis and pericarditis were disproportionally reported following the BNT162b2 mRNA vaccine when compared with both the Ad26.COV2.S and mRNA-1273 vaccines, independently of the disproportionality measure used in the analysis (Figure 2). The results of the sensitivity analysis performed on cases with a validated diagnosis of myocarditis and pericarditis by laboratory analyses or imaging were consistent with those provided in the main analysis (Figure 3).

## 4. Discussion

In this comprehensive analysis based on VAERS, we found reporting rates of myocarditis and pericarditis to be less than 0.1% for all three types of COVID-19 vaccines authorized for use in the US. Reporting rates were highest for the BNT162b2 mRNA vaccine, followed by the mRNA-1273 and Ad26.COV2.S vaccines, respectively. Almost half of the cases were confirmed by laboratory testing and/or imaging without the presence of other risk factors for myocarditis or pericarditis.

The first report of myocarditis following administration of the BNT162b2-mRNA vaccine described 62 individuals in Israel, primarily young men (25). Two of these cases were fatal. Since then, reports also emerged from the US where the incidence of myocarditis or pericarditis after COVID-19 vaccination was higher than expected when compared with background rates (6, 26). The fact that most cases were seen in previously healthy male adolescents or young men, and generally developed within a week of vaccine administration, agrees with prior reports (27–31). Interestingly, rates of myocarditis or pericarditis after administration of the BNT162b2-mRNA and mRNA-1273 vaccines appear to be an order of magnitude higher than those previously described for the smallpox vaccine among military personnel (32). Conversely, rates seem similar for the Ad26.COV2.S vaccine.

The risk of myocarditis or pericarditis following COVID-19 vaccination is likely lower than the risk of these conditions secondary to COVID-19 infection (33). Moreover, cases of COVID-19-related myopericarditis are likely underreported (34). One review reported a 15-fold higher risk of myocarditis or pericarditis after SARS-CoV-2 infection (150–4,000 cases/100,000 individuals) compared with pre-COVID levels (1–10 cases/100,000 individuals) (35). Finally, a recent systematic review and meta-analysis found a more than 7-fold higher in persons who were infected with the SARS-CoV-2 than in those who were vaccinated (36).

Our study extends data from prior studies that used data from VAERS (11–16). For example, Alami et al. found the combined rate of myocarditis and pericarditis to be higher after the second vaccine dose and highest among males aged 12–17 years (∼6 cases per 100,000 s doses) (11). Reporting rates were similar between both mRNA COVID-19 vaccines (BNT162b2-mRNA and mRNA-1273) across the different age groups. These results were corroborated by a comprehensive study by Oster et al. (14) Laurini et al. also found disproportionality for BNT162b2 as compared with mRNA-1273 for myocarditis (15). Finally, Woo et al. raised a potential safety concern for the Ad26.COV2.S vaccine with respect to myocarditis (16).

In most prior reports on COVID-19 vaccine-related myocarditis and pericarditis, the diagnosis was based on laboratory testing and noninvasive imaging. However, a case series of two patients demonstrated histologically confirmed myocarditis after the BNT162b2-mRNA and mRNA-1273 vaccines (37). In both these cases, an inflammatory infiltrate with macrophages, *T*-cells, eosinophils, and B-cells was seen upon endomyocardial sampling. Testing for viral genomes or autoantibodies in the tissue specimens was not performed, but no other causes were identified by polymerase chain reaction or serologic examination. Therefore, although causality between the COVID-19 vaccines and these cardiovascular complications remains unproven, it does appear likely because of the temporal association and the lack of other plausible causes (6).

The systemic inflammatory response to immunizations can lead to both myocardial and pericardial inflammation (38). The underlying mechanism has not been fully elucidated, but is most likely nonspecific innate inflammation or molecular mimicry, leading to eosinophilic hypersensitivity myocarditis as has been seen for other drugs and vaccines (38). Indeed, the presentation is fairly similar to that of idiopathic myopericarditis (27–32, 39). While some studies have reported abnormal left ventricular systolic function in this setting (29–31), long-term prognostic implications are unknown. It is also unclear why more cases have been reported after the mRNA-based vaccines. This may be due to their different mechanism of action, the fact that two doses are administered, or because the BNT162b2-mRNA vaccine is more commonly used in younger individuals who may be more susceptible to develop myocarditis and pericarditis.

### 4.1. Limitations

Our results should be considered in virtue of the limitations of VAERS. Spontaneous reporting systems are susceptible to both underreporting and incomplete reporting. Accordingly, it is not possible to accurately assess the frequency or incidence of adverse events using these data sources. As an example, there was a significantly higher incidence of myocarditis and pericarditis after small-pox vaccination when actively following patients compared with passive case finding (38). Spontaneous reporting databases are also sensitive to the high variability of data quality without adjudication, differential reporting, and the lack of an accurate denominator. These factors may influence the reliability of head-to-head comparisons among vaccines (40, 41).

Furthermore, it is important to acknowledge the emergence of bivalent versions of the COVID-19 vaccines, such as the d26.COV2.S, BNT162b2 mRNA, and mRNA-1273 SARS-CoV-2 vaccines. While our analysis focused on the safety of existing vaccine formulations, it is crucial to recognize that this study did not include an evaluation of the bivalent versions as they were not available at the time of our analysis. Future research should consider examining the effectiveness and potential benefits of these specific vaccines, as they may contribute to a more comprehensive understanding of the evolving landscape of COVID-19 vaccination strategies. Including such analysis could provide valuable insights into the comparative safety profiles of different vaccine formulations.

## 5. Conclusions

We found reporting rates of myocarditis and pericarditis to be less than 0.1% after COVID-19 vaccination. Rates were highest for the BNT162b2 mRNA vaccine, followed by the mRNA-1273 and Ad26.COV2.S vaccines, respectively. The reporting rates of cardiovascular complications such as myocarditis and pericarditis secondary to vaccination remain less common than those seen for SARS-CoV-2 infection.

## Data Availability

Publicly available datasets were analyzed in this study. This data can be found here: https://vaers.hhs.gov/.

## References

[1] Adler Y Charron P Imazio M Badano L Barón-Esquivias G Bogaert J 2015 ESC guidelines for the diagnosis and management of pericardial diseases: the task force for the diagnosis and management of pericardial diseases of the European Society of Cardiology (ESC)Endorsed by: the European association for cardio-thoracic sur. Eur Heart J. (2015) 36:2921–64. 10.1093/eurheartj/ehv318 26320112 PMC7539677

[2] LeWinter MM. Clinical practice. Acute pericarditis. N Engl J Med. (2014) 371:2410–6. 10.1056/NEJMcp1404070 25517707

[3] Sagar S Liu PP Cooper LTJ. Myocarditis. Lancet (London, England). (2012) 379:738–47. 10.1016/S0140-6736(11)60648-X 22185868 PMC5814111

[4] Imazio M Brucato A Barbieri A Ferroni F Maestroni S Ligabue G Good prognosis for pericarditis with and without myocardial involvement: results from a multicenter, prospective cohort study. Circulation. (2013) 128:42–9. 10.1161/CIRCULATIONAHA.113.001531 23709669

[5] Siripanthon B Nazarian S Muser D Deo R Santangeli P Khanji MY Recognizing COVID-19-related myocarditis: the possible pathophysiology and proposed guideline for diagnosis and management. Hear Rhythm. (2020) 17:1463–71. 10.1016/j.hrthm.2020.05.001 PMC7199677 32387246

[6] Wise J. COVID-19: should we be worried about reports of myocarditis and pericarditis after mRNA vaccines? Br Med J. (2021) 373:n1635. 10.1136/bmj.n1635 34167952

[7] Sadoff J Le Gars M Shukarev G Heerwegh D Truyers C de Groot AM Interim results of a phase 1–2a trial of Ad26.COV2.S COVID-19 vaccine. N Engl J Med. (2021) 384:1824–35. 10.1056/NEJMoa2034201 33440088 PMC7821985

[8] Baden LR El Sahly HM Essink B Kotloff K Frey S Novak R Efficacy and safety of the mRNA-1273 SARS-CoV-2 vaccine. N Engl J Med. (2021) 384:403–16. 10.1056/NEJMoa2035389 33378609 PMC7787219

[9] Polack FP Thomas SJ Kithin N Absalon J Gurtman A Lockhart S Safety and efficacy of the BNT162b2 mRNA COVID-19 vaccine. N Engl J Med. (2020) 383:2603–15. 10.1056/NEJMoa2034577 33301246 PMC7745181

[10] Shay DK Shimabukuro TT DeStefano F. Myocarditis occurring after immunization with mRNA-based COVID-19 vaccines. JAMA Cardiol. (2021) 6(10):1115–7. 10.1001/jamacardio.2021.2821 34185047

[11] Alami A Krewski D Mattison D Wilson K Gravel CA Villeneuve PJ Risk of myocarditis and pericarditis among young adults following mRNA COVID-19 vaccinations. Vaccines (Basel). (2022) 10:722. 10.3390/vaccines10050722 35632478 PMC9147275

[12] Chen C Fu F Ding L Fang J Xiao J. Booster dose of COVID-19 mRNA vaccine does not increase risks of myocarditis and pericarditis compared with primary vaccination: new insights from the vaccine adverse event reporting system. Front Immunol. (2022) 13:938322. 10.3389/fimmu.2022.938322 36172346 PMC9510366

[13] Hatziantoniou S Anastassopoulou C Lazaros G Vasileiou K Tsioufis C Tsakris A. Comparative assessment of myocarditis and pericarditis reporting rates related to mRNA COVID-19 vaccines in Europe and the United States. Expert Rev Vaccines. (2022) 21:1691–6. 10.1080/14760584.2022.2100765 35815358

[14] Oster ME Shay DK Su JR Gee J Creech CB Broder KR Myocarditis cases reported after mRNA-based COVID-19 vaccination in the US from December 2020 to August 2021. JAMA. (2022) 327:331–40. 10.1001/jama.2021.24110 35076665 PMC8790664

[15] Laurini GS Montanaro N Broccoli M Bonaldo G Motola D. Real-life safety profile of mRNA vaccines for COVID-19: an analysis of VAERS database. Vaccine. (2023) 41:2879–86. 10.1016/j.vaccine.2023.03.054 37024412 PMC10043970

[16] Woo EJ Gee J Marquez P Baggs J Abara WE McNeil MM Post-authorization safety surveillance of ad.26.COV2.S vaccine: reports to the vaccine adverse event reporting system and v-safe, February 2021–February 2022. Vaccine. (2023) 41:4422–30. 10.1016/j.vaccine.2023.06.023 37321898 PMC10264169

[17] Wu K. Myocarditis. BMJ best Pract. (2021):1. Available at: https://bestpractice.bmj.com/topics/en-gb/244

[18] Baruah R. Pericarditis. BMJ best Pract. (2021):1. Available at: https://dev.bp-frontend.tf.aws.bmjgroup.com/topics/en-gb/3000214

[19] Pilero A Agostinelli C Sessa M Quaglino P Santucci M Tomasini C Langerhans, plasmacytoid dendritic and myeloid-derived suppressor cell levels in mycosis fungoides vary according to the stage of the disease. Virchows Arch. (2017) 470(5):575–82. 10.1007/s00428-017-2107-1 28321511

[20] Sessa M Sportiello L Mascolo A Scavone C Gallipoli S di Mauro G Campania preventability assessment committee (Italy): a focus on the preventability of non-steroidal anti-inflammatory drugs’ adverse drug reactions. Front. Pharmacol. (2017) 8:305. 10.3389/fphar.2017.00305 28603499 PMC5445158

[21] Sessa M Sullo MG Mascolo A Cimmaruta D Romano F Puca RV A case of figurate urticaria by etanercept. J Pharmacol Pharmacother. (2016) 7:106–8. 10.4103/0976-500X.184777 27440958 PMC4936077

[22] Sessa M Rossi C Mascolo A Grassi E Fiorentino S Scavone C Suspected adverse reactions to contrast media in campania region (Italy): results from 14 years of post-marketing surveillance. Expert Opin Drug Saf. (2015) 14:1341–51. 10.1517/14740338.2015.1067301 26156557

[23] Sessa M Rafaniello C Sportiello L Mascolo A Scavone C Maccariello A Campania region (Italy) spontaneous reporting system and preventability assessment through a case-by-case approach: a pilot study on psychotropic drugs. Expert Opin Drug Saf. (2016) 15:9–15. 10.1080/14740338.2016.1221397 27875917

[24] Sessa M Rossi C Rafaniello C Mascolo A Cimmaruta D Scavone C Campania preventability assessment committee: a focus on the preventability of the contrast media adverse drug reactions. Expert Opin Drug Saf. (2016) 15:51–9. 10.1080/14740338.2016.1226280 27855534

[25] Reuters. Israel Examining heart inflammation cases in people who received pfizer COVID shot. Internet. (2021) 1. Available at: https://www.reuters.com/world/middle-east/israel-examining-heart-inflammation-cases-people-who-received-pfizer-covid-shot-2021-04-25/

[26] Gubernot D Jazwa A Niu M Baumblatt J Gee J Moro P U.S. population-based background incidence rates of medical conditions for use in safety assessment of COVID-19 vaccines. Vaccine. (2021) 39:3666–77. 10.1016/j.vaccine.2021.05.016 34088506 PMC8118666

[27] Mouch SA Roguin A Hellou E Ishai A Shoshan U Mahamid L Myocarditis following COVID-19 mRNA vaccination. Vaccine. (2021) 39:3790–3. 10.1016/j.vaccine.2021.05.087 34092429 PMC8162819

[28] Muthukumar A Narasimhan M Li Q Mahimainathan L Hitto I Fuda F In-depth evaluation of a case of presumed myocarditis after the second dose of COVID-19 mRNA vaccine. Circulation. (2021) 144:487–98. 10.1161/CIRCULATIONAHA.121.056038 34133883 PMC8340727

[29] Larson KF Ammirati E Adler ED Cooper LT Jr Hong KN Saponara G Myocarditis after BNT162b2 and mRNA-1273 vaccination. Circulation. (2021) 144:506–8. 10.1161/CIRCULATIONAHA.121.055913 34133884 PMC8340725

[30] Rosner CM Genovese L Tehrani BN Atkins M Bakhshi H Chaudhri S Myocarditis temporally associated with COVID-19 vaccination. Circulation. (2021) 144:502–5. 10.1161/CIRCULATIONAHA.121.055891 34133885 PMC8340723

[31] Dickey JB Albert E Badr M Laraja KM Sena LM Gerson DS A series of patients with myocarditis following SARS-CoV-2 vaccination with mRNA-1279 and BNT162b2. JACC. Cardiovasc. Imaging. (2021) 14:1862–3. 10.1016/j.jcmg.2021.06.003 34246585 PMC8219373

[32] Halsell JS Riddle JR Atwood E Gardner P Shope R Poland GA Myopericarditis following smallpox vaccination among vaccinia-naive US military personnel. JAMA. (2003) 289:3283–9. 10.1001/jama.289.24.3283 12824210

[33] Ling RR Ramanathan K Tan FL Tai BC Somani J Fisher D Myopericarditis following COVID-19 vaccination and non-COVID-19 vaccination: a systematic review and meta-analysis. Lancet Respir Med. (2022) 10:679–88. 10.1016/S2213-2600(22)00059-5 35421376 PMC9000914

[34] Kornowski R Witberg G. Acute myocarditis caused by COVID-19 disease and following COVID-19 vaccination. Open Heart. (2022) 9:e001957. 10.1136/openhrt-2021-001957 35264415 PMC8914394

[35] Fairweather D Beetler DJ Di Florio DN Musigk N Heidecker B Cooper LT Jr. COVID-19, myocarditis and pericarditis. Circ Res. (2023) 132:1302–19. 10.1161/CIRCRESAHA.123.321878 37167363 PMC10171304

[36] Voleti N Reddy SP Ssentengo P. Myocarditis in SARS-CoV-2 infection vs. COVID-19 vaccination: a systematic review and meta-analysis. Front Cardiovasc Med. (2022) 9:951314. 10.3389/fcvm.2022.951314 36105535 PMC9467278

[37] Verma AK Lavine KJ Lin C-Y. Myocarditis after COVID-19 mRNA vaccination. N. Engl. J. Med. (2021) 385(14):1332–4. 10.1056/NEJMc2109975 34407340 PMC8385564

[38] Engler RJM Nelson MR Collins L Jr Spooner C Hemann B Gibbs BT A prospective study of the incidence of myocarditis/pericarditis and new onset cardiac symptoms following smallpox and influenza vaccination. PLoS One. (2015) 10:e0118283. 10.1371/journal.pone.0118283 25793705 PMC4368609

[39] Montgomery J Ryan M Engler R Hoffman D McClenathan B Collins L Myocarditis following immunization with mRNA COVID-19 vaccines in members of the US military. JAMA Cardiol. (2021) 6(10):1202–6. 10.1001/jamacardio.2021.2833 34185045 PMC8243257

[40] Alomar M Tawfiq AM Hassan N Palaian S. Post marketing surveillance of suspected adverse drug reactions through spontaneous reporting: current status, challenges and the future. Ther Adv Drug Saf. (2020) 11:2042098620938595. 10.1177/2042098620938595 32843958 PMC7418468

[41] Mascolo A Scavone C Sessa M di Mauro G Cimmaruta D Orlando V Can causality assessment fulfill the new European definition of adverse drug reaction? A review of methods used in spontaneous reporting. Pharmacol Res. (2017) 123. 10.1016/j.phrs.2017.07.005 28694146

